# Supplementary figures and images for: A LAMP sequencing approach for high-throughput co-detection of SARS-CoV-2 and influenza virus in human saliva
Source: eLife. 2022 May 9;11:e69949. doi: 10.7554/eLife.69949 (PMC9084890; doi:10.7554/eLife.69949)

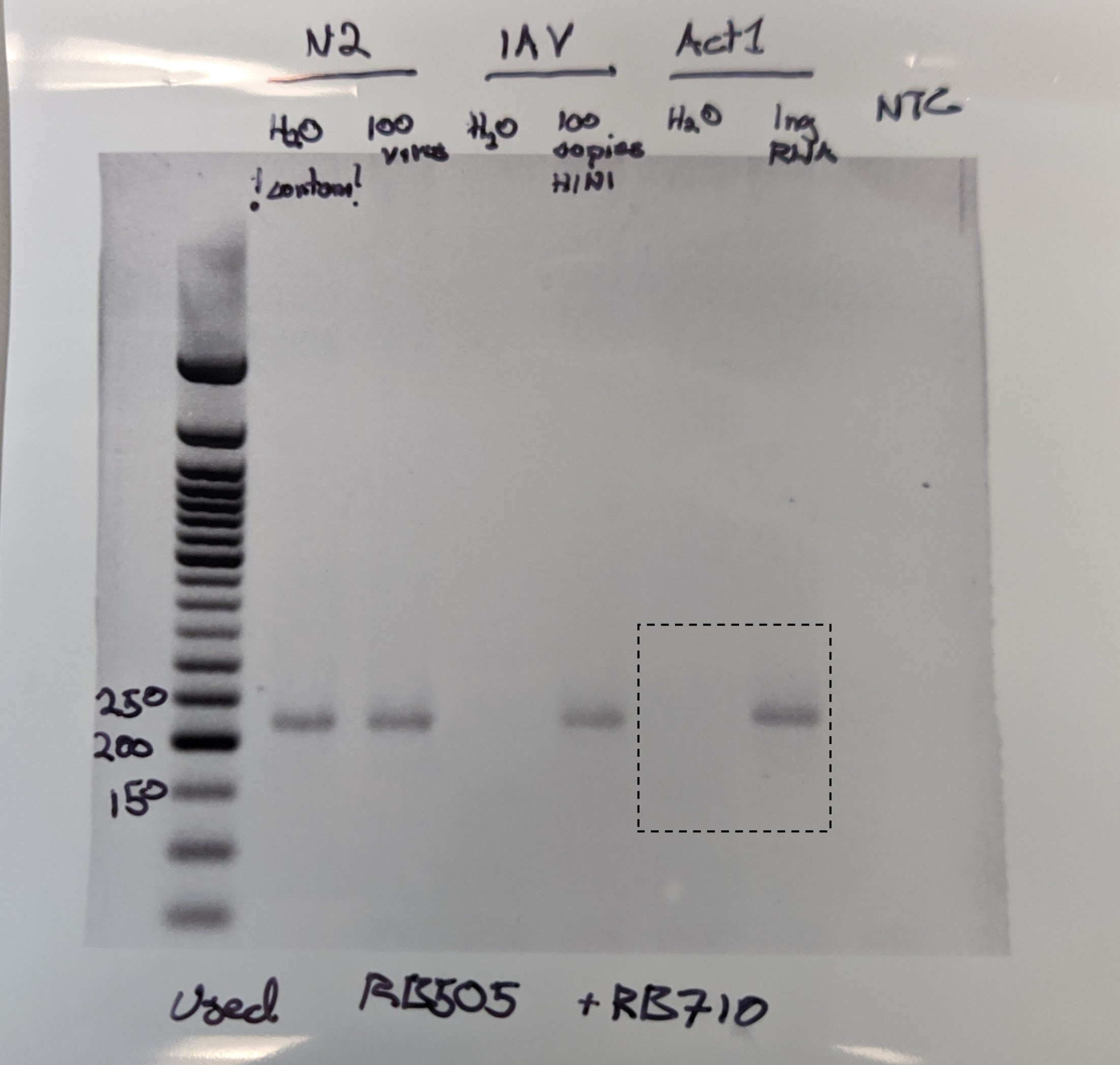

Supplement: Figure 1—source data 1. [file elife-69949-fig1-data1.png]

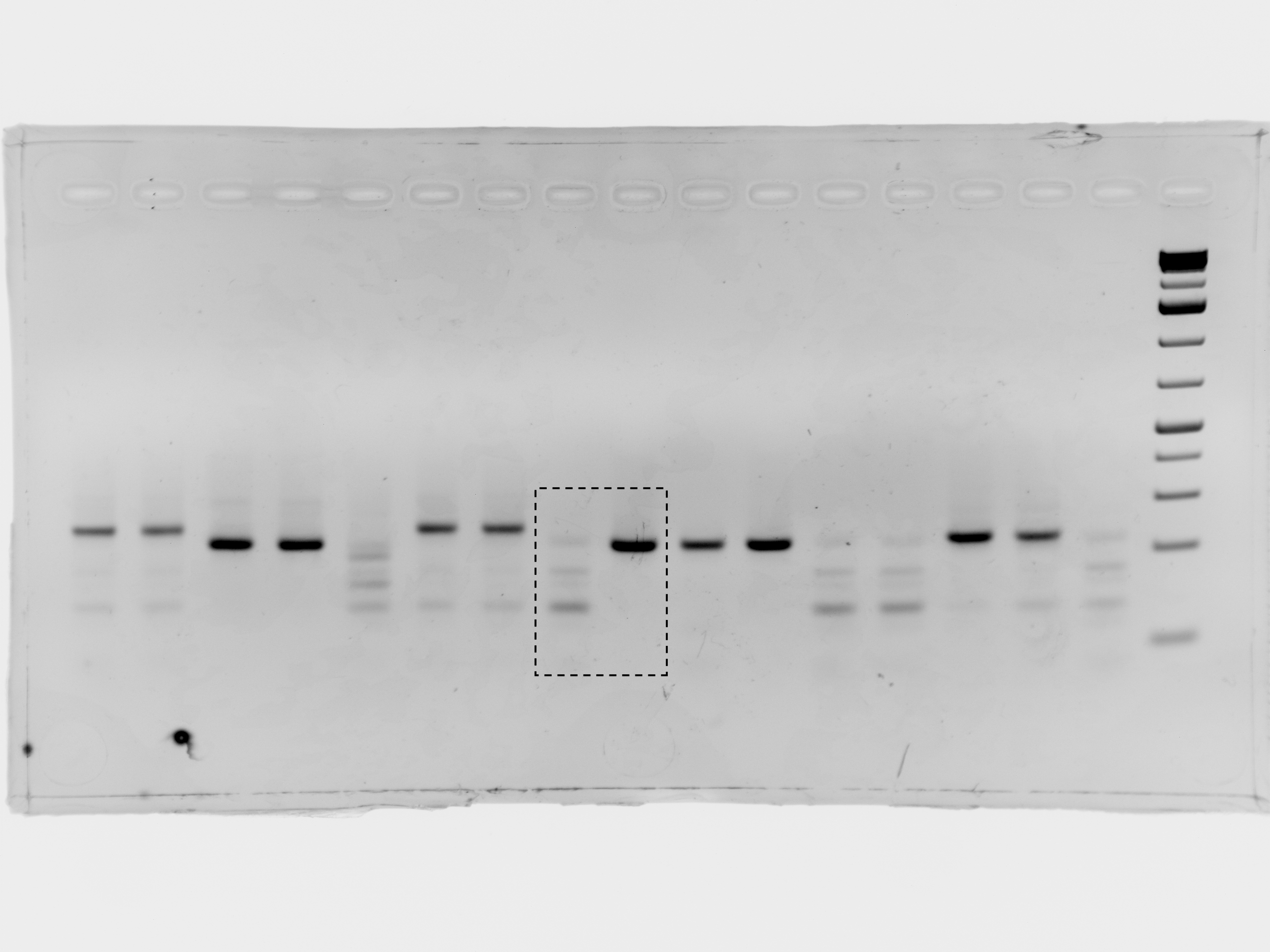

Supplement: Figure 2—source data 1. [file elife-69949-fig2-data1.png]

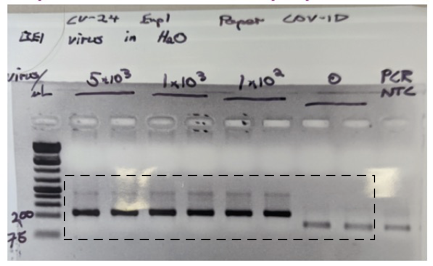

Supplement: Figure 4—source data 1. [file elife-69949-fig4-data1.png]

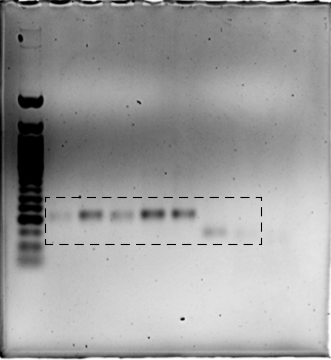

Supplement: Figure 4—source data 2. [file elife-69949-fig4-data2.png]
